# Depression among Medical Students of a Medical College in Nepal during COVID-19 Pandemic: A Descriptive Cross-sectional Study

**DOI:** 10.31729/jnma.5441

**Published:** 2021-07-31

**Authors:** Aarshee Adhikari, Ezna Sujakhu, GC Sandervee, Sabin Bahadur Zoowa

**Affiliations:** 1 Nepalese Army Institute of Health Sciences, Sanobharyang, Kathmandu, Nepal; 2 Department of Community Medicine, Nepalese Army Institute of Health Sciences, Sanobharyang, Kathmandu, Nepal

**Keywords:** *COVID-19*, *depression*, *medical students*, *mental health*, *Nepal*

## Abstract

**Introduction:**

Depression is a common mental disorder. Medical school is a stressful environment. The outbreak of COVID-19 has added to the plight of medical students. This study was conducted to determine the prevalence of depression among medical students of a medical college in Nepal during the COVID-19 pandemic.

**Methods:**

A descriptive cross-sectional study was conducted among medical students of a medical college in Nepal from August 2020 to September 2020. The sample size of our study was 223. A convenient sampling method was adopted for the selection of respondents. The study was approved by the Institutional Review Committee (Reference no:321). The data were analyzed using Statistical Package for Social Sciences version 22. Point estimate at 95% Confidence Interval was calculated along with frequency and proportion for binary data. The study instrument consisted of the Patient Health Questionnaire and socio-demographic information.

**Results:**

Out of 223 participants, the prevalence of depression was seen among 52 (23.3%) (17.7-28.9 at 95% Confidence Interval) students in our study. Among them, 25 (48.1%) females and 27 (51.9%) males were depressed. The prevalence of depression was higher in preclinical years (first and second year) than in clinical years (third, fourth and final year).

**Conclusions:**

The prevalence of depression among medical students in Nepal during the pandemic was less than the findings of similar studies conducted in Nepal before the pandemic. Further studies are required to get more knowledge about the factors associated with mental health of medical students.

## INTRODUCTION

Depression is a common mental disorder affecting more than 264 million people worldwide.^1^ Studies have found that the prevalence of depression among medical students is higher than depression in the general population.^2^ Medical school is a stressful environment.^3^ Moreover, the outbreak of the 2019 novel coronavirus (COVID-19) has added to the plight of medical students.

It is important to understand the mental health of medical students, especially during the COVID-1 9 pandemic. This will prevent the consequences of poor mental health by addressing the root of the problem and implementing effective preventive programs. There is a lack of adequate studies on the mental health of medical students in Nepal.^3^ Moreover, no similar studies have been conducted during COVID-1 9 as far as we know.

The main aim of this study was to determine the prevalence of depression among medical students of a medical college during the COVID-19 pandemic.

## METHODS

This descriptive cross-sectional study was conducted at Nepalese Army Institute of Health Sciences-College of Medicine (NAIHS-COM) from August 2020 to September 2020. The total number of students in the academic course was 537. Using the Cochran formula for sample size calculation for finite samples, our sample size was 223 students.

The sample size was calculated by using formula,

n_0_ = Z^2^ × p × q / e^2^

  = (1.96)^2^ × (0.5) × (1-0.5) / (0.05)^2^

  = 384.16

Where,

n_0_ = the recommended sample size
Z = 1,96 at 95% Confidence Interval
p = 50% (proportion or percentage or prevalence of an attribute)
q = 1-p
e = margin of error i.e. 5%

Sample size was adjusted for finite population

n = n_0_/1+ (n_0_ - 1)/N

  = 384.16 / [1 + (384.16 - 1) / 537)]

  = 222.80

  = 223

Where,

n = the new adjusted sample size
N = the population size, 537 (the total number of medical students of our medical school)

Convenient sampling method was adopted for the selection of respondents. The study was approved by the Institutional Review Committee, Nepalese Army Institute of Health Sciences, (Ref. no: 321). A letter of approval was taken from NAIHS-COM for the data collection. The email addresses of all the students attending NAIHS-COM were collected. A set of selfadministered Google form questionnaires containing questions regarding socio-demographic factors, personal and family information, PHQ-9 and informed consent was sent. For data collection, the aim of the study was briefly described to participants. The different factors and variables were studied.

The Patient Health Questionnaire (PHQ-9) is a depression module, which requires students to rate each of the 9 depressive symptoms as 'not at all', 'several days', 'more than half the days' and 'nearly every day'. Using PHQ-9 modules, these ratings were assigned scores of 0, 1, 2, and 3 respectively. A cut-off score of 5, 10, 15, and 20 signifies mild, moderate, moderately severe, and severe depression, respectively. A score of ≥10 has a sensitivity of 88% and a specificity of 88% for major depression.^4^ In this study, students scoring ≥10 were considered having depression.

The data were analyzed using Statistical Package for Social Sciences (SPSS) version 22.

## RESULTS

**Figure 1:**
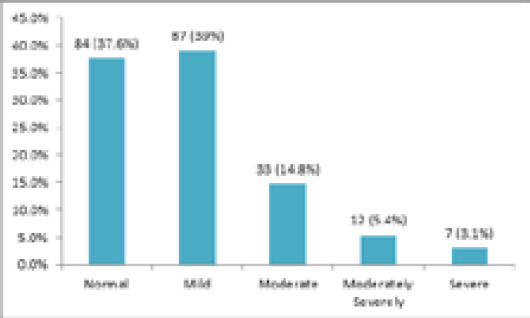
Prevalence of depression among medical students of NAIHS-COM.

The prevalence of depression among medical students in our study was among 52 (23.3%) (17.7-28.9 at 95% Confidence Interval). The students who scored 0-4, 5-9, 10-14, 15-19, 20-27 in PHQ-9 questionnaire were considered normal, mildly depressed, moderately depressed, moderately severely depressed, and severely depressed, respectively. As per the scores, among total participants, 84 (37.6%) were normal, 87 (39%) were mildly depressed, 33 (14.8%) were moderately depressed, 12 (5.4%) were moderately severely depressed, and 7 (3.1%) were severely depressed (Figure 1).

Among the 223 participants, there were 135 (60.5%) males and 88 (39.5%) females (Table 1).

**Table 1:** Socio-demographic information (n= 223).

Profile	Description	n (%)
	18-20	86 (38.6)
Age	21-23	116 (52.0)
	24-26	21 (9.4)
Sex	Male	135 (60.5)
	Female	88 (39.5)
	1st year	68 (30.5)
	2nd Year	48 (21.5)
Study Level (MBBS)	3rd year	52 (23.3)
	4th year	26 (11.7)
	5th year	29 (13.0)

Out of the 52 depressed medical students, 25 (48.1%) were females and 27 (51.9%) were males (Table 2).

**Table 2:** Gender wise prevalence of depression.

Gender	n (%)
Female	25 (48.1)
Male	27 (51.9)

**Figure 2:**
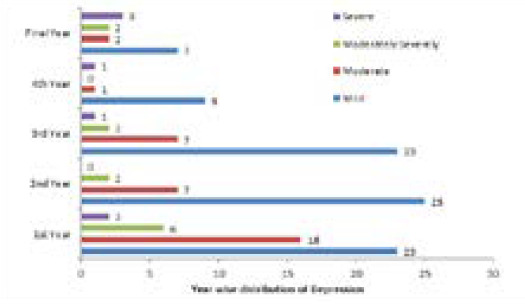
Distribution of severity of depression among different year of medical students.

The rate of depression in first, second, third, fourth and final year students were 24 (46.1%), 9 (17.3%), 10 (19.2%), 2 (3.8%) and 7 (13.4%), respectively (Figure 2).

In preclinical years (first and second year), 33 (63.4%) were depressed whereas, in clinical years (third, fourth and final year), 19 (36.5%) were depressed.

In the age groups 18-20, 21-23 and 24-26, 23 (44.2%), 23 (44.2%) and 6 (11.5%) were depressed respectively (Table 3).

**Table 3:** Age wise prevalence of depression (n= 52).

Age	n (%)
18-20	23 (44.2)
21-23	23 (44.2)
24-26	6 (11.5)

## DISCUSSION

In our study, the prevalence of depression among medical students during this pandemic was found to be 23.3%. According to a study conducted in China among university students during the COVID-19 pandemic the prevalence of depression was 23.3% which is similar to our findings.^5^ In similar studies done in Nepal before the pandemic by Shrestha N, et al. and Basnet B, et al. the prevalence of depression was 27.20% and 29.78% respectively.^6,7^ This decrease might be because of the nationwide lockdown done by the government in response to the COVID-19 pandemic due to which all the medical schools remained closed. Hence, the academic workload and stressors might have decreased compared to before the pandemic. Most of the students were at home with their family which might have contributed positively to this decrease as well.

According to our study, depression among female students was 48.1% and male students was 51.9%. Similar studies conducted in Nepal showed the prevalence of depression higher in females 32% than in males 28%.^6,7^ The trend of depression being more prevalent among females than among males in the general population has been seen in different studies.^8^ When females and males are confronted with similar stressors, females have greater reactivity and may be more vulnerable than males to develop depression due to factors like biological responses, self-concepts, and coping styles.^9^

Our findings also show depression among students of the age group 24-26 years to be 11.5% followed by those of 21-23 years and 18-20 years to be 44.2% each. According to our findings, depression was found to be more among first-year students (46.1%). This finding is similar to that of studies done in Portugal, which showed a higher prevalence of depression in first year medical students.^10^ Similarly, in a study done by Adhikari A, et al. the prevalence of depression was higher in preclinical students than in clinical students.^11^ In Nepal, students enrol in medical colleges just after graduating high school. The challenge of a new environment, as well as the stress of increased academic workload after joining the medical college, might be the reason for the higher depression rate in first-year medical students. The high rate of depression among final year students (13.4%) is perhaps due to the academic burnout caused by preparations for major subject examinations for their final year.

There were several limitations to our study. Since our study only included students from a single medical college, the findings of this study cannot be generalized to all medical students. This was a convenience type of sampling, so there was no set of criteria for participant inclusion which could have led to bias in the result. In addition to this, as the participants were asked to fill the questionnaire via Google form, many of them might not have had access to the internet, which could have led to non-response bias resulting in a small sample size. Some participants may have felt pressured to give socially acceptable answers leading to response bias.

## CONCLUSIONS

The prevalence of the depression among medical students in our study during the pandemic was found to be less than the findings of similar studies conducted in Nepal before the pandemic. There should be an increase in the number of studies conducted to assess the mental health problems of medical students in Nepal. Each medical college should include a student counselling unit that will help the students cope with their problems. Moreover, during adverse times as the COVID-19 pandemic, there should be good cooperation between medical colleges, students and parents to facilitate the development of the students.
